# Use of low frequency ultrasound for water treatment: Data on azithromycin removal

**DOI:** 10.1016/j.dib.2020.105947

**Published:** 2020-06-27

**Authors:** Adrián Muñoz-Calderón, Henry Zúñiga-Benítez, Sergio H. Valencia, Ainhoa Rubio-Clemente, Sergio A Upegui, Gustavo A. Peñuela

**Affiliations:** a Grupo GDCON, Facultad de Ingeniería, Sede de Investigación Universitaria (SIU), Universidad de Antioquia UdeA, Calle 70 # 52 -21, Medellín, Colombia; b Departamento de Ingeniería Química, Facultad de Ingeniería, Universidad de Antioquia UdeA, Calle 70 # 52-21, Medellín, Colombia; c Facultad de Ingeniería, Tecnológico de Antioquia Institución Universitaria, Calle 78B # 72A-220, Medellín, Colombia

**Keywords:** Advanced oxidation technologies, Antibiotics, Azithromycin, Ultrasound, Wastewater treatment

## Abstract

Azithromycin (AZT) is a broad-spectrum antibiotic present in different aqueous matrices due to its incomplete removal using conventional water treatments. Ultrasound (US) is an advanced oxidation technology that has demonstrated its capacity to degrade different types of organic molecules due to the generation of cavitation bubbles or cavities that promote the generation of radicals.

In this paper, data regarding the use of low-frequency US (40 kHz) in the removal of AZT are presented. Tests were carried out at lab scale for 60 min considering a reaction volume of 300 mL (pollutant initial concentration 1.0 mg *L*^−1^). The effect of operational parameters such as pH, ultrasound power, the presence of external agents like ferrous ions, hydrogen peroxide, and UV radiation were evaluated. In general, obtained data show that under the experimental reaction conditions, it is feasible to reach extents of AZT removal ∼50.0%, and that the presence of other species in the medium could inhibit the reaction, mainly due to scavenging effects. This information is relevant to future applications of US, at pilot or real scale, in the treatment of water with presence of AZT or similar organic pollutants.

**Specifications table**

**Table:** 

**Subject**	Chemical Engineering
**Specific subject area**	Advanced oxidation technologies
**Type of data**	Figure
**How data were acquired**	Data were obtained using Ultra High-Performance Liquid Chromatography (UHPLC).
	Microsoft Excel was employed for figures preparation.
**Data format**	Raw
	Analysed
**Parameters for data collection**	Data were collected at fixed experimental conditions. Effects of operational parameters such as solution pH, ultrasound power, the presence of external agents like ferrous ions, hydrogen peroxide, and UV radiation were evaluated.
	US frequency and AZT initial concentration were constant during all the tests (40 kHZ and 1.0 mg *L* ^−^ ^1^).
**Description of data collection**	Tests were carried out at lab conditions. Reaction temperature was controlled and fixed at 25.0 °C. Reaction time was 60 min, and samples were collected at different time intervals.
	Initial pH, AZT concentration, and presence of external agents were monitored during the experiments.
	Results were analysed considering the extent of pollutant elimination (difference between initial concentration and final or evaluated concentration).
**Data source location**	Grupo Diagnóstico y Control de la Contaminación (GDCON), Engineering College, Universidad de Antioquia (UdeA), Medellín-Colombia.
**Data accessibility**	Data are available only in this article and the associated supplementary file.

**Value of the data**

• Data show that low-frequency (40 kHZ) ultrasound could be appropriate to remove the antibiotic azithromycin from aqueous solutions.
• Data could benefit researches, and academic, public or private institutions working on the application of advanced oxidation technologies on water treatment.
• Data could be used in the planification of experimental conditions to evaluate the potential application of ultrasound or other advanced oxidation technologies on azithromycin or similar organic pollutants elimination.
• Data could be used in the development of methodologies for azithromycin removal at pilot or real scale.
• Data include the effect of the solution initial pH, the ultrasound power, and the presence of external agents such as ferrous ions, hydrogen peroxide, and UV radiation, which is relevant in terms of design and implementation of wastewater treatment processes.

## 1 Data description

Data presented in this work describe the azithromycin (AZT) removal using low-frequency ultrasound (US). US is an advanced oxidation technology, in which hydroxyl radical species (OH•) are generated after the formation and implosion of cavitation bubbles as it is indicated by Eq. (1) [1]. OH• free radicals are able to react with organic pollutants faster than other oxidizing agents.

(1) H_2_O +))) → H• + OH•

This work includes data regarding the use of 40 kHZ US on AZT removal considering the effect of different operational parameters. In this way, Table 1 shows the tests carried out and the evaluated factors.

**Table 1:** Evaluated experimental conditions.

Test	pH	US power (W)	H_2_O_2_ concentration (mg *L* ^−^ ^1^)	Fe^+2^ concentration (mg *L* ^−^ ^1^)	UV ligth radiation
**1**	3.0	50.0	—	—	—
**2**	7.0	50.0	—	—	—
**3**	9.0	50.0	—	—	—
**4**	9.0	16.5	—	—	—
**5**	9.0	25.0	—	—	—
**6**	9.0	41.5	—	—	—
**7**	9.0	50.0	2.4	—	—
**8**	9.0	50.0	4.8	—	—
**9**	9.0	50.0	7.2	—	—
**10**	9.0	50.0	—	0.5	—
**11**	9.0	50.0	—	2.5	—
**12**	9.0	50.0	—	5.0	—
**13**	9.0	50.0	—	—	**+**
**14**	9.0	50.0	—	—	**+**
**15**	9.0	50.0	—	—	**+**

**Fig. 1:**
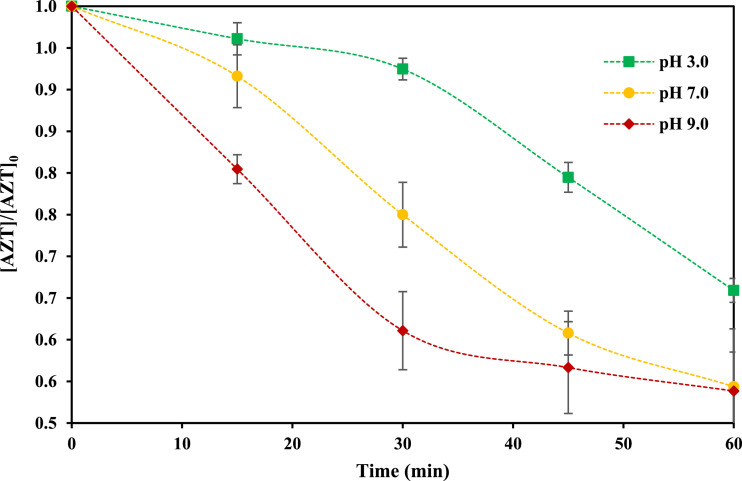
Effect of solution pH on AZT removal using US (AZT initial concentration 1.0 mg *L*^−1^, power 50.0 W, frequency 40 kHz, temperature 25.0 ± 2.0 °C, reaction volume 300 mL).

Fig. 1 presents the results associated with the AZT removal under different conditions of pH. From the Figure, it can be appreciated that higher pH values promote a higher AZT removal. This tendency could be correlated to the molecular charge of the pollutant and its distribution in the solution. AZT is a not volatile compound, which means that its reaction with generated radicals would be in the bulk solution, or in the cavitation bubble-liquid interface [2]. pKa of AZT is 8.96 [3], therefore under lower pH conditions the molecule is charged positive (more solubility), and under pH > 8.96, AZT is in its neutral form (less solubility).

**Fig. 2:**
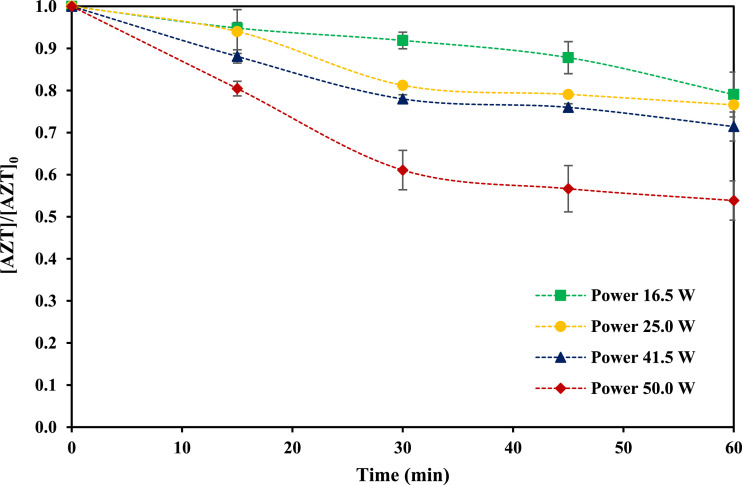
Effect of applied power on AZT removal using US (AZT initial concentration 1.0 mg *L*^−1^, pH 9.0, frequency 40 kHz, temperature 25.0 ± 2.0 °C, reaction volume 300 mL).

Effect of applied US power on AZT removal was studied considering four experimental levels (16.5, 25.0, 41.5 and 50.0 W). Solution pH was fixed at 9.0. Fig. 2 presents the obtained data, which indicate that a higher power promotes a higher pollutant removal. In this case, increasing the US power could imply a higher formation of cavitation bubbles, and then more OH• free radicals. However, it has been reported that a very high power, could generate a negative effect on organic compounds elimination using US as a result of an oversaturation of bubbles in the solution, and less implosion [4].

**Fig. 3:**
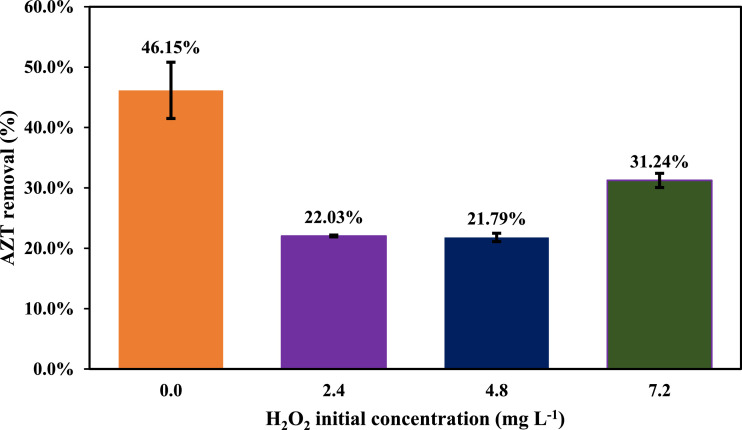
Effect of H_2_O_2_ on AZT removal using US (AZT initial concentration 1.0 mg *L*^−1^, pH 9.0, power 50.0 W, frequency 40 kHz, temperature 25.0 ± 2.0 °C, reaction volume 300 mL).

Effect of H_2_O_2_ presence in the reaction was evaluated under three experimental levels 2.4, 4.8 y 7.2 mg *L*^−1^. Adding H_2_O_2_ to reactions involving US could promote organic pollutants degradation. US is able to break the H_2_O_2_ molecule generating additional hydroxyl radicals (Eq. (2)) [5]. However, Fig. 3 shows that the presence of peroxide inhibits the AZT removal. This result would be associated with the fact that an excess of H_2_O_2_ could trap OH• radicals (Eq. (3)) [6], reducing an eventual antibiotic elimination.

(2) H_2_O_2_ +))) → 2 OH•

(3) H_2_O_2_ + OH• → HO_2_• + H_2_O

One of the parallel reactions that occurs during ultrasonic treatments is the recombination of OH• radicals to produce H_2_O_2_ (Eq. (4)), which implies the accumulation of peroxide in the system, and a possible inhibition on AZT removal. In this way, the addition of ferrous ions could reduce this effect, since Fe^2+^reacts with H_2_O_2_ to generate additional OH• (Fenton reaction, Eq. (5)) [7].

(4) OH• + OH• → H_2_O_2_

(5) H_2_O_2_ + Fe^2+^→ OH• + Fe^3+^+ OH^−^

**Fig. 4:**
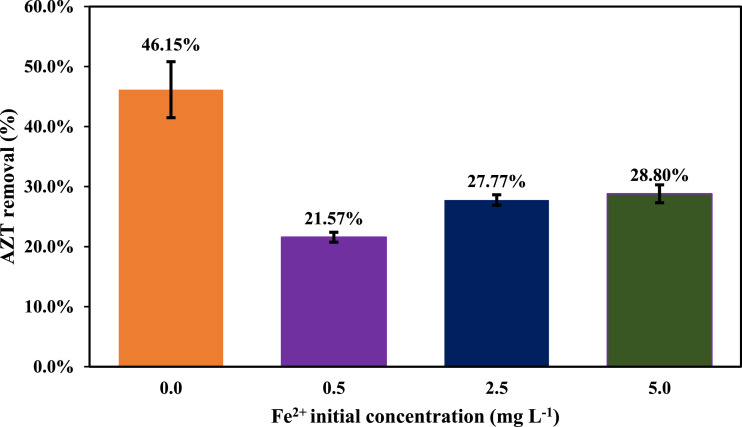
Effect of Fe^2+^ on AZT removal using US (AZT initial concentration 1.0 mg *L*^−1^, pH 9.0, power 50.0 W, frequency 40 kHz, temperature 25.0 ± 2.0 °C, reaction volume 300 mL).

The effect of adding Fe^+2^ on the reaction was evaluated considering three experimental levels (0.5, 2.5 and 5.0 mg *L*^−1^). Fig. 4 indicates that Fe^+2^ induces an inhibitory effect on the reaction, a situation that could be related to a scavenging effect associated with Fe^2+^ excess. In addition, it has been reported that reactions involving Fe^2+^ and H_2_O_2_ require low acidic pH ((3), (4), (5)) conditions, and that under a basic pH, Fe^2+^ solubility is limited [8]. In this way, future studies should evaluate this aspect and its effect on AZT removal using low frequency US.

**Fig. 5:**
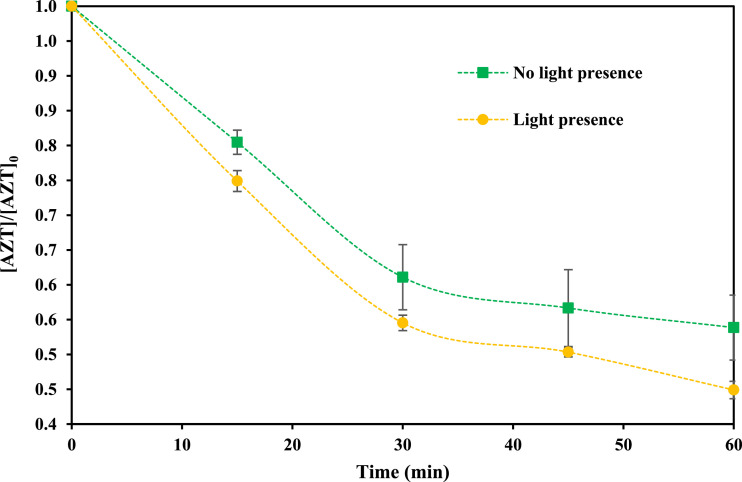
Effect of UV light on AZT removal using US (AZT initial concentration 1.0 mg *L*^−1^, pH 9.0, power 50.0 W, frequency 40 kHz, temperature 25.0 ± 2.0 °C, reaction volume 300 mL).

Finally, Fig. 5 shows the data about the effect of UV light presence on sono-treatment. The incorporation of UV could promote the generation of additional radicals because of the H_2_O_2_ molecule decomposition. In addition, light in the UV range alone has the capacity to broke different organic compounds [9,10].

Supplementary data file associated with this article contains the data employed to created Fig. 1, Fig. 2, Fig. 3, Fig. 4, Fig. 5, including results of each experiment, its average value, and data standard deviations.

## 2 Experimental design, materials and methods

Experiments were carried out using a 40 kHz ultrasonic transducer (Meinhardt Ultrasonics) with variable power between 0.0 and 50.0 W. The transducer was coupled to the bottom of a glass reactor with a maximum capacity of 500 mL.

Used chemicals were of analytical grade. AZT dihydrate (98% purity) was supplied by AK Scientific. FeSO_4_·7H_2_O (Sigma Aldrich) was used as Fe^2+^ ions source, and H_2_O_2_ (35% w/w) was supplied by Merck. pH was adjusted using NaOH and HCl concentrated solutions obtained from Alfa-Aesar.

AZT initial concentration in all the tests was 1.0 mg *L*^−1^, and solutions were prepared using ultra-pure deionized water as solvent. In this way, extent of pollutant removal was determine comparing the variation of AZT concentration with the initial value.

AZT concentration quantification and monitoring were done using Ultra-High-Performance Liquid Chromatography (UHPLC). An Acquity UPLC system (Waters Corporation) and an Acquity UPLC BEH C18 column (1.7 mm, 50 mm, 2.1 mm, Waters) were employed. Mobile phase was a mix of water: methanol (95:5, aqueous phase) and methanol: water (95:5, organic phase). Aqueous/organic phases ratio was 60/40 for one min, then 40/60 for one additional min, next 10/90 for one min; and finally, 40/60 for two minutes. Masslynx 4.1 (Micromass) software was used to process data, and injection volume was 10 μL.

All the tests were done in triplicate.

## Declaration of Competing Interest

The authors declare that they have no known competing financial interests or personal relationships which have, or could be perceived to have, influenced the work reported in this article.

## Appendix Supplementary materials

Image, application 1

Image, application 2
